# A genetic algorithm-Bayesian network approach for the analysis of metabolomics and spectroscopic data: application to the rapid identification of Bacillus spores and classification of Bacillus species

**DOI:** 10.1186/1471-2105-12-33

**Published:** 2011-01-26

**Authors:** Elon Correa, Royston Goodacre

**Affiliations:** 1 School of Chemistry, The University of Manchester, 131 Princess Street, Manchester, M1 7ND, UK; 2 Manchester Centre for Integrative Systems Biology, Manchester Interdisciplinary Biocentre, University of Manchester, 131 Princess Street, Manchester, M1 7ND, UK

## Abstract

**Background:**

The rapid identification of *Bacillus *spores and bacterial identification are paramount because of their implications in food poisoning, pathogenesis and their use as potential biowarfare agents. Many automated analytical techniques such as Curie-point pyrolysis mass spectrometry (Py-MS) have been used to identify bacterial spores giving use to large amounts of analytical data. This high number of features makes interpretation of the data extremely difficult We analysed Py-MS data from 36 different strains of aerobic endospore-forming bacteria encompassing seven different species. These bacteria were grown axenically on nutrient agar and vegetative biomass and spores were analyzed by Curie-point Py-MS.

**Results:**

We develop a novel genetic algorithm-Bayesian network algorithm that accurately identifies sand selects a small subset of key relevant mass spectra (biomarkers) to be further analysed. Once identified, this subset of relevant biomarkers was then used to identify *Bacillus *spores successfully and to identify *Bacillus *species via a Bayesian network model specifically built for this reduced set of features.

**Conclusions:**

This final compact Bayesian network classification model is parsimonious, computationally fast to run and its graphical visualization allows easy interpretation of the probabilistic relationships among selected biomarkers. In addition, we compare the features selected by the genetic algorithm-Bayesian network approach with the features selected by partial least squares-discriminant analysis (PLS-DA). The classification accuracy results show that the set of features selected by the GA-BN is far superior to PLS-DA.

## Background

*Bacillus *and *Clostridium *species can adapt to rapidly changing environments and starvation by developing spores. An endospore is a dormant non-reproductive structure produced by these Gram-positive bacteria and is a survival mechanism adapted to spending a long period of time in hostile conditions. The sporulation process in *Bacillus *species causes singular molecular and cellular changes in the cell which are not seen in the vegetative state [1]. One of these changes is the biosynthesis of calcium dipicolinate, which is found in sporulated cells but not in the vegetative ones.

Members of the genus *Bacillus *are widely distributed in the environment and because their spores are so resistant their control is of considerable importance in the food manufacture [2]. Some of these bacteria are pathogenic including *B. cereus *and *B. subtilis *which cause food poisoning. The most notorious member of this genus is *B. anthracis*, which is the causal agent of anthrax, and the rapid identification of spores and bacterial identification are paramount because of its importance as a potential biological warfare agent [3] and in bioterrorism [4]. Thus there is a need to have a generic characterisation method that allows rapid identification of spores and typing of bacteria.

Many automated analytical techniques such as Raman spectroscopy [5], liquid and gas chromatography [6,7] and Curie-point pyrolysis mass spectrometry (Py-MS) [8-10] have been used to identify bacterial spores. All of these methods rely on chemometric analyses of the data and the question arises as to how robust these mathematical models are. However, the vast majority of modelling approaches are considered "black boxes" as they do not readily allow the specific association between input analytical data and output classification to be revealed.

These types of data analysis involve a large number of features to be analysed, such as several mass spectra. This high number of features makes interpretation of the data extremely difficult Therefore, we start our data analysis by reducing data dimensionality. This data reduction step selects a small subset of key relevant masses to be further analysed and discards the less important ones. This feature selection procedure uses Bayesian network learning methods coupled with genetic algorithms to identify bacterial spores and classify *Bacillus *species.

A Bayesian network (BN) is basically a graphical model of a probability distribution over a set of variables of a given problem domain [11,12]. This graphical model provides a compact and intuitive representation of the relationships among variables of a given problem domain. Nodes on the graph represent variables from the problem (e.g., *m/z *intensities) and an arrow linking two nodes indicates a statistical correlation between them. This statistical correlation falls broadly into one of the two categories: (a) "positive correlation" indicates that the values of both variables increase or decrease together, and (b) "negative correlation" indicates that as one variable increases, the other decreases, or *vice versa*. The network structure of a BN encodes probabilistic dependencies among domain variables and a joint probability distribution quantities the strength of these dependencies [13]. The resulting graphical model or network has two main uses. (1) Visualization of probabilistic relationships: the graphical model provides direct and accurate information about the underlying interactions among variables of interest, *m/z *intensities in our case, and (2) Inference: the Bayesian network is intrinsically an inference model and can be used to predict outputs or to classify new samples.

We use statistical and data mining algorithms to identify *Bacillus *spores automatically from their Curie-point pyrolysis mass spectrometry data. This process extends the data mining analysis to a two step hierarchical-based classification that further identifies the bacilli into one of their respective species. First, the data dimensionality is reduced by a feature selection process using genetic algorithms (GA) and BNs in parallel. Subsequently, once that the relevant variables are identified, a classification model using only BN is built based on the reduced data set, and this process undergoes full validation. Next a statistical analysis of the interactions among variables and classes and variables and variables is performed using the built Bayesian network model. As this combined process identifies *probabilistic *biomarkers in the data set it is possible to develop predictive, testable models that allow inference of biological properties of the bacilli. The computer code for the GA-BN algorithm developed on this work was written in R programming language [14] ver. 2.9.2 and is freely available, together with the *Bacillus *data set, on request to the corresponding author.

## Methods

### The *Bacillus *data set

The work uses the *Bacillus *Py-MS data set reported in [8] and described as an additional file (see Additional file 1). Unlike most data sets which concentrate on a single or only a handful of *Bacillus *species, this data set investigates 36 different strains of aerobic endospore-forming bacteria encompassing seven different species: *Bacillus amyloliquefaciens*, *Bacillus cereus*, *Bacillus licheniformis*, *Bacillus megaterium*, *Bacillus subtilis (including Bacillus niger and Bacillus globigii), Bacillus sphaericus*, and *Brevibacillus laterosporus*. These bacteria were grown axenically on nutrient agar as detailed in [8,15] and vegetative biomass and spores were analyzed in triplicates by Curie-point Py-MS. The data set contains 216 *Bacillus *samples; 108 are vegetative and 108 are sporulated. For a more detailed explanation of this data set see [8]. A phylogenetic tree of the type strains of the *Bacillus *species studied in this paper can be found in [16].

## Data analysis

**Figure 1:**
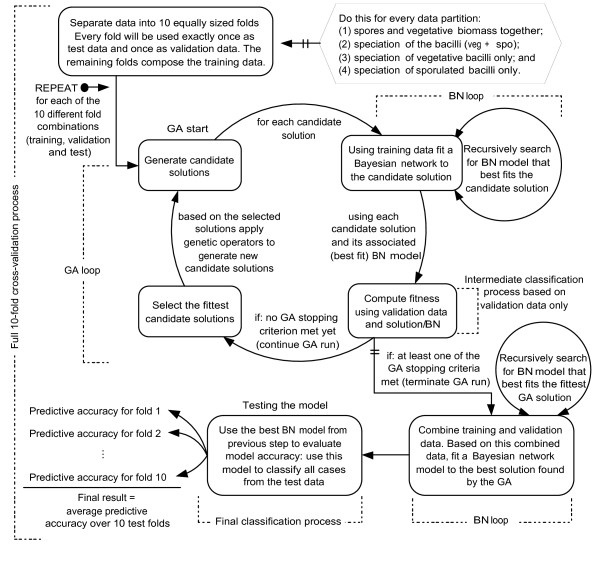
**Work flow for the data analysis**. A genetic algorithm is used for feature selection. The GA's fitness function is the predictive accuracy, on the validation data, given by Bayesian network model built for each individual candidate solution. At the end of the GA run, a new BN model is built for the best GA solution using the combined training and validation data. This new BN model is then tested on the previously untouched test data. The classification results described in this paper are the average predictive accuracy over 10 test folds.

The overall work flow for the data analysis is shown in Figure 1 and involved a two stage process. The input data sets for the data analysis contained the full Py-MS spectra, 150 *m/z *intensities. The data were normalised as a percentage of the total ion count to remove the influence of sample size *per se*.

**Stage 1 **employed a genetic algorithm for feature selection with classification of (a) either spores *versus *vegetative biomass or (b) speciation to one of the seven different species. The classifier used is the Bayesian network that best fits the best solution (with a reduced set of features) found by a genetic algorithm. In this supervised learning procedure, the measurement of the fitness of a GA solution in this study follows a wrapper approach. The wrapper approach searches for an optimal feature subset tailored to a particular algorithm, such as a Bayesian network. For more information on wrapper and other feature selection approaches see [17].

**Stage 2 **involved the fitting of a new Bayesian network model to the best GA solution found on the previous stage. The built BN model is then used to determine probabilistic relationships between the *m/z *intensities selected by the GA and the classification (sporulation status or speciation). This two step process and model validation are detailed below.

### Genetic algorithms (GAs)

A GA is an optimization procedure that evolves a population of candidate solutions to solve an objective function [18]. A GA repeatedly applies operators based on natural selection and genetic recombination to the candidate solutions. In a standard GA the initial solutions are randomly generated using a uniform distribution. The candidate solutions are called chromosomes. The chromosomes are usually represented by fixed-length strings over a finite alphabet. The term fitness is used to describe the quality of a candidate solution. The fitness is a measurement of how well the chromosome solves the objective function. The fitness associated with a chromosome is used to select probabilistically which chromosomes from the population will recombine and possibly generate new solutions. A genetic operator called crossover is applied to create two new chromosomes (progeny) from a pair of selected chromosomes called parents. The crossover consists of swapping random subsets of the genetic material from both parents. Because of the selective pressure applied on the population through a number of generations, the overall trend is towards higher-fitness chromosomes. Mutations are used to help preserve diversity in the population by introducing random changes into the chromosomes. Both crossover and mutation are usually applied with user-defined probabilities, and in general, the probability of crossover is much larger than the probability of mutation. For more details on genetic algorithms see [19-21]. The design of our genetic algorithm is as follows. We employed a binary chromosome with 150 bits, one for each *m/z *variable. A gene value of 1 indicates that the corresponding m/z variable is selected and a value of 0 indicates that it is not selected. A population size of 200 candidate solutions was used, with a crossover and mutation probabilities of 0.75 and 0.033, respectively. During the test phase of the GA these parameters generated the best results; but we make no claim that they are optimal parameter values. Setting the parameters of a GA is not a trivial task and GA parameter optimization is a topic for future investigation. The objective function that we employed was to maximize classification accuracy. The fitness of a candidate solution was assessed as the classification accuracy, on a validation set, achieved by the BN model built for the candidate solution being assessed. As each candidate solution represents a different subset of features (*m/z *intensities), the BN model built for a particular solution is a classification model based solely on the features present on that solution, e.i., the features that correspond to the genes whose value is equal to 1 inside the chromosome, Figure 1. The stopping criteria used were: (1) maximum of 200 generations are performed on a single GA run, (2) a solution whose classification model (BN) produces 100% of predictive accuracy is found, or (3) when all the 200 solutions in the population converge to a single solution.

### Bayesian networks (BNs)

A BN is a graphical map of the probabilistic relationships among variables of a given problem domain [22]. The graphical representation of a BN is a directed acyclic graph. A directed acyclic graph *G *is an ordered pair *G *= *(V; E) *where V is a set whose elements are called vertices or nodes and *E *is a set whose elements are called directed edges, arcs, or arrows. The graph *G *contains no directed cycles; for any vertex υ ∈ *V *, there is no directed path that starts and ends on υ. An example of a Bayesian network is as follows. This is a modified version of the so-called "Asia" problem found in [23]. Suppose that a doctor is treating a patient who has been suffering from shortness of breath, called dyspnoea. The doctor knows that diseases such as tuberculosis, bronchitis and lung cancer are possible causes of this. The doctor also knows that other relevant information includes whether the patient is a smoker, which increases the chances of lung cancer and bronchitis, and what sort of air pollution the patient has been exposed to. A positive X-ray would indicate either tuberculosis or lung cancer. The set of variables for this problem and their possible values are shown in an additional file (see Additional file 2) together with a Bayesian network representing this problem. The network structure, also known as network topology, shows how variables correlate to each other. More formally, let **X **= {*X*_1_, *X*_2_,..., *X_n_*} be a multivariate random variable whose components *X_i _*are also random variables. A corresponding lower-case letter *x_i _*denotes an assignment of state or value to the random variable. *X_i _Parents *(*X_i_) *represents the set of nodes that have a directed edge pointing to *X_i_*.

Consider a BN containing *n *nodes, *X_1 _*to *X_n_*, taken in that order. A particular value of **X **= {*X*_1_, *X*_2_,..., *X_n_*} in the joint probability distribution is represented by *p*(**X**) = *p*(X_1 _= *x*_1_, X_2 _= *x*_2_, ..., X*_n _*= *x_n_*), or more compactly, *p*(*x*_1_, *x*_2_, ..., *x_n_*). The chain rule of probability theory allows the factorization of joint probabilities, therefore:

(1) $$\begin{array}{l} p(\text{X})=p(x_{1})p(x_{2}|x_{1})...p(x_{n}|x_{1},\;...,\;x_{n-1})\\ \;\;\;=\prod_{i}p(x_{i}|x_{1},\;...,\;x_{i-1}). \end{array}$$

As the structure of a BN implies that the value of a particular node is conditional only on the values of its parent nodes, Equation 1 is reduced to:

(2) $$p(\text{X})=\prod_{i}p(X_{i}|Parents(X_{i})).$$

Learning the structure of a BN is an NP-hard problem [24,25]. Many algorithms developed to this end use a scoring metric and a search procedure. The scoring metric evaluates the "goodness-of-fit" of a structure to the data. The search procedure generates alternative structures and selects the best one based on the scoring metric. A greedy search algorithm is used to generate alternative structures for the BN. Greedy search algorithms make a locally optimal choice at each stage. Starting with an empty network, the greedy search algorithm adds to the current network the edge that most increases the score of the new resulting network. The search stops when no other edge addition improves the score of the network. Additional file 3 shows the pseudocode of a generic greedy search algorithm for learning Bayesian network structures. In this paper, we use an unconventional scoring metric to evaluate the goodness-of-fit" (score) of a network structure to the data. The scoring metric adopted is specific to our classification task and is computed as follows. The entire data set is divided into mutually exclusive training and test sets, see "Model validation" for more details. The training set is further divided into two mutually exclusive parts - training and validation sets. The first part (training set) is used to compute the probabilities for the Bayesian network. The second part is used as the validation set. During the search for the best possible network structure only the validation set is used to compute predictive accuracy and the measurement (quality) of the predictive accuracy achieved is used as the scoring metric for the Bayesian network model proposed. The higher the predictive accuracy value, the better the BN model fits the data. Once the best GA solution is found, at the end of the GA run, the validation set and the training set are merged and this merged data (i.e., the entire original training set) is used to compute the probabilities for a new BN fitting to this data. Using this newly built BN model the predicted accuracy, for that combination of folds, is then computed on the previously untouched test set. The results reported in this paper are the average predictive accuracy over 10 entirely distinct test folds.

To summarize, the Bayesian network is used both in parallel to the GA and sequentially after each complete GA run is performed as shown in Figure 1. **First, the parallel GA-BN usage**. For each new GA solution the following procedure is repeated. Solution *S_k _*is generated. A Bayesian network model *B_k _*is built based only on the features (*m/z *variables) that are selected in *S_k _*and using the training set to compute probabilities. The model *B_k _*is then used to predict the samples on the validation set and the predictive accuracy produced by *B_k _*becomes the fitness value of solution *S_k_*, i.e., fitness value of *S_k _*= predictive accuracy resulting from model B_k_. At the end of the GA run, the solution with the highest fitness value *S*_best _is retained. **Second, the sequential BN usage after the complete GA-BN run**. A new Bayesian network *B*_best _is built based on *S*_best _but this time using the combined data, training set + validation set, for computation of probabilities. The predictive accuracy of *B*_best _is then evaluated on a third, previously untouched fold, the test set. This result is then added to the computation of the average accuracy over the 10-fold cross-validation. The process is repeated for each of the 10 folds.

### Feature selection

Many data mining applications involve the task of building a model. The goal of such a model is to classify data records according to a set of common features. Feature selection, also known as variable or attribute selection, is the technique of selecting a subset of relevant variables for building robust and accurate learning models. In the present work, feature selection using GA is used to reduce data dimensionality before a classification model is built. The reduction of the number of variables speeds up model building and improves model stability and interpretability. In addition, we independently applied a partial least squares-discriminant analysis [26,27] (PLS-DA) algorithm for feature selection. PLS-DA is a particular case of partial least squares regression where the dependent variable, or response, is a binary or a dummy variable. We use the PLS-DA implementation form the R package "Classification and Regression Training" (Caret) ver. 4.31 written by Max Kuhn [28]. The importance of every variable for the prediction model is measured by their variable importance for projection (VIP) coefficient. Variables with large VIP, larger than 1, are the most relevant ones for the model and contribute more for class discrimination.

### Model validation

For an accurate measurement of model generalization, ideally, model validation would involve the prediction of classes on a completely new and independently generated data set addressing the same questions under investigation. In practice, however, those two complete and independently generated sets of data are rarely available and the researcher has to balance the trade-off between model overfitting and generalization using the unique set of data available. To deal with this problem our model validation uses a standard data mining procedure called cross-validation [17,29]. First, the complete data set is divided into 10 equally sized folds. The folds are randomly generated but under the following criteria: (1) the proportion of classes in every single fold has to be similar to the proportion of classes found in the original data set containing all records (this is known as stratified cross-validation, and importantly (2) replicates are kept together and not split into different folds. Each of the 10 folds is used once as a test set. Out of the 9 remaining folds, 8 folds are used as training set and 1 fold is reserved to be a validation set. The Bayesian network uses the training set (8 folds) to compute the probabilities required to classify new examples. Once those probabilities are computed, the BN classifies the examples in the validation set. When the run of the GA algorithm is completed, the training set (8 folds) and the validation set (1 fold) are merged into a full, bigger training set. This full merged training set is used to re-compute the probabilities for a new BN fitted to the best solution (subset of features) returned by the GA. The new Bayesian network is then used to classify examples in the test set (the 10th fold), which was never accessed before during the run of the genetic algorithm. The reasons for having separate validation and test sets are that in a classification model the goal is to measure predictive accuracy (generalization ability) on a test set unseen during training. Hence, the test set cannot be used by the GA, and is reserved just to compute the predictive accuracy associated with the Bayesian network built with the best set of variables selected at the end of the GA run.

## Results and Discussion

### Discriminating between different physiological states

First, a GA was used for the variable selection phase as detailed above. Thirty independent runs of the genetic algorithm were performed. Each individual run included a 10-fold cross-validation, therefore 300 randomly generated folds were analyzed overall. All variables that appeared in 70% or more of the final GA solutions were selected, and this included 17, out of the 150 variables: *m/z *58, *m/z *59, *m/z *60, *m/z *63, *m/z *64, *m/z *65, *m/z *70, *m/z *76, *m/z *77, *m/z *79, *m/z *84, *m/z *90, *m/z *92, *m/z *99, *m/z *105, *m/z *132 and *m/z *154. We chose a cutoff percentage of 70% for two main reasons: (1) to simplify the interpretation of the Bayesian network model by limiting the number of variables used to build the model, and (2) to concentrate the analysis only on the most relevant features from the data. After this variable selection two classification models were built using Bayesian networks. The first BN model is built using all 150 variables available; the second BN model is built using only the 17 GA-selected variables. Table 1 shows the confusion matrices for both classification models and the statistics related to those matrices. TP and FP are the numbers of true positives and false positives respectively.

**Table 1:** Vegetative and sporulated classes: confusion matrices and summary statistics, w. avg. = weighted average.

Confusion Matrices					
		**predicted class**			
		
		**all 150 *m*/*z *intensities**		**17 GA selected *m*/*z *intensities**	
			
		**veg**	**spo**	**veg**	**spo**
actual class	veg spo	108 1	0 107	108 1	0 107

Statistics					

class		TP	FP	precision	Sensitivity

veg spo		1 0.995	0.009 0	0.991 1	1 0.991

w. avg.		0.995	0.005	0.995	0.995

**Figure 2:**
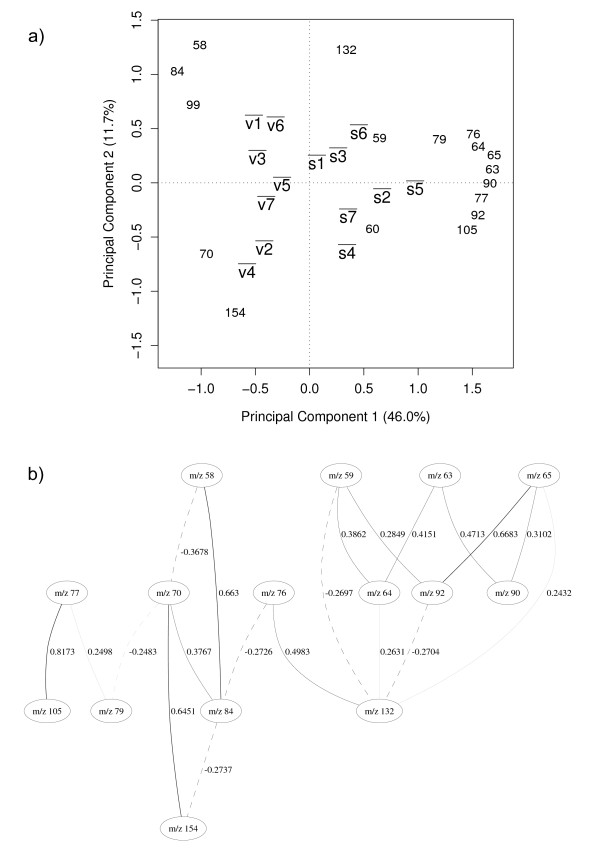
**Physiological states**. (a) principal component analysis. $\bar{\text{v}}=$ vegetative, $\bar{\text{s}}\;\text{=}$ sporulated, $\bar{1}=$*amyloliquefaciens*, $\bar{2}=$*cereus*, $\bar{3}=$*licheniformis*, $\bar{4}=$*megaterium*, $\bar{5}=$*sphaericus*, $\bar{6}=$*subtilis*, $\bar{7}=$*laterosporus*. Discriminating between vegetative and sporulated classes. Physiological states are labelled as $\text{"}\bar{\text{v}}\text{"}$ for vegetative and $\text{"}\bar{\text{s}}\text{"}$ for sporulated. Even though the species are not yet considered on this analysis, numbers (from 1 to 7) are shown beside the physiological state to indicate species type (see labels on the graph). The bar over the labels indicates that each point represents an average over all similar cases on the data set. $\bar{\text{v}}$ and $\bar{\text{s}}$ represent group centers for PC scores and the numbers (which represent *m/z *intensities) are PC loadings. (b) Bayesian network model. Solid lines indicate a positive correlation between nodes, dashed lines indicate negative correlation and the thickness of the line indicates the strength of the correlation; therefore the thicker the line is the stronger the correlation. The number beside the line shows the partial correlation coefficient for that correlation.

The results in Table 1 show an identical predictive accuracy for the classifications with either all 150 variables or the reduced data (17 GA selected variables). In practice the classification model using only 17 variables should be favoured because it is less computationally expensive to run, more parsimonious [30], and also simpler to interpret. Whilst the variable selection and classification model shows the most relevant variables for the identification of *Bacillus *spores, it does not give any information about how these variables correlate to the classes or indeed to each other. Therefore, we use principal component analysis (PCA) to detect groups of *m/z *intensities that strongly correlate to each other and observe their relationships to the physiological state of the bacilli. Figure 2(a) shows the biplot for the average PC scores value of the 14 classes and overlaid on this plot are the *m/z *intensities from the PC loadings matrix.

The PC scores plot in Figure 2(a) shows a clear separation between the vegetative $\left(\bar{\text{v}}\right)$ and sporulated $\left(\bar{s}\right)$ bacilli in the first PC which accounts for 46% of the total explained variance. The *m/z *intensities that appear to the right of the vertical dotted line that divides the graph have a strong correlation with the sporulated physiological state and the intensities to the left of that line are strongly correlated to the vegetative state. The proximity of $\bar{\text{v}1}$ and $\bar{\text{v6}}$ on the graph indicates that in their vegetative state *B*. *amyloliquefaciens *and *B. subtilis *are very closely related and this reflects the known taxonomy of these two species [16]; these are also closely related to the taxonomically similar *B. licheniformis *$\left(\bar{\text{v}3}\right)$. This is also seen in the spores of these bacteria. This suggests that despite choosing *m/z *that can discriminate between spores and vegetative cells some information on the different species is still present. This is also true for *B. cereus *$\left(\bar{\text{v2}}\right)$ and *B. megaterium *$\left(\bar{\text{v4}}\right)$ that also co-cluster and are taxonomically similar [16]. To examine the interactions among the *m/z *intensities further we have developed a powerful probabilistic model based on Bayesian networks. Contrary to intuition, the direction of the arrows in a Bayesian network does not necessarily imply a cause-effect relationship between the variables; that is to say a Bayesian network is not a "causal network". Thus to eliminate possible confusion we have intentionally omitted the arrow heads from our BN graphs. Figure 2(b) shows the BN describing the association of the 17 selected *m/z *intensities, and as detailed above this was fully validated using 10-fold cross-validation. Solid lines indicate a positive correlation between nodes, dashed lines indicate negative correlation and the thickness of the line indicates the strength of the correlation. Therefore the thicker the line is the stronger the correlation. The number beside the line shows the partial correlation coefficient for that correlation. A partial correlation estimates the correlation between two nodes when the effect of all other nodes in the model is held constant, and this process avoids finding variables (*m/z *) that are not directly correlated to each other [31].

The network Figure 2(b) shows the correlation among the *m/z *intensities. The strongest correlation on the network is a positive correlation between *m/z *105 and *m/z 77*. *m/z *105 is a pyridine ketonium ion known to arise from DPA [8] and Beverly *et al.*, Havey *et al*. and Opitz have suggested that *m/z *77 results from the electron ionization fragmentation pathway via loss of CO from *m/z *105 [32-34]. As these *m/z *are highly correlated in the BN and appear closer to the sporulated classes on Figure 2(a), the results indicate that for sporulated bacilli the *m/z *105 and *m/z *77 intensities are noticeably higher than for the vegetative case.

In order to generate a rule for differentiating spores from vegetative cells we also applied a classification and regression trees algorithm (CART) implemented according to the methods described in [35] and written in R programming language [14]. The classification tree procedure creates a tree-based classification model which classifies cases into groups. The procedure provides validation tools for exploratory and confirmatory classification analysis. The CART algorithm produced a classification tree containing only four biomarkers, *m/z *63, *m/z *77, *m/z *84 and *m/z *105, as sufficient to discriminate accurately between the physiological states of the bacilli. We then proceeded to use discriminant analysis to produce a classification equation using those four m/z intensities. To compute such equation we used the discriminant analysis option in the software "Statistical Package for the Social Sciences" SPSS Inc. [36] ver. 16. The equation coefficients are canonical discriminant coefficients. The resulting formula is shown in Equation 3.

(3) $$f(MZ)=\{\begin{array}{cc} \text{vegetative,} & \text{if }h\;\leq \text{ 0,}\\ \text{spore,} & \text{otherwise}\text{.} \end{array}$$

where *h *= 3.523 *m/z 63 *+ 4.193 *m/z 77 - *1.007 *m/z 84 - *0.347 *m/z *105 *- *0.961, and *MZ *= (*m*/z *63*, *m/z 77*, *m/z 84 *; *m/z 105*). This equation successfully classifies 100% of the cases from the *Bacillus *data set correctly. Thus we have understood some of the relationships between the variables, confirmed that DPA and its product ions in Electron Impact Mass Spectrometry (EI-MS) are excellent biomarkers for spores and have a very parsimonious model where only 4 of the original 150 ions are used to describe the solution adequately.

### Discriminating between species

For this analysis the species are considered as classes (*n *= 7). In order to ascertain whether there was any difference in classifications based on physiology, we performed these experiments using three different partitions of the mass spectral data. The first partition uses all the 216 bacilli collectively, vegetative + sporulated. The second partition uses only vegetative cells and the third one uses only sporulated cells. Experiments on the second and third partitions expect that one has already differentiated between the two physiology states (as illustrated above) and would work like a hierarchical classification were the physiological state of the cases is known and only the species have to be predicted.

**Figure 3:**
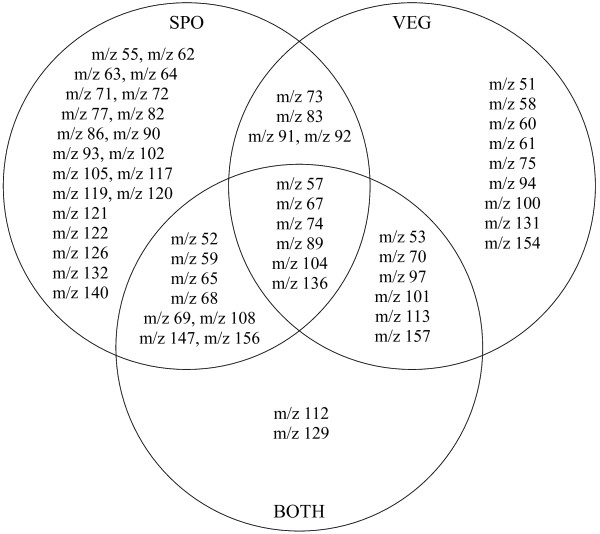
**Venn diagram**. Variables selected for different partitions of the *Bacillus *data set. BOTH = results from the speciation of all bacilli (including vegetative and sporulated cases); VEG = results from the speciation of vegetative bacilli only; and SPO = results from the speciation of sporulated bacilli only.

The variable selection GA-classifier was applied to each of the three partitions of the data and produced the subsets of variables shown in the Venn diagram Figure 3.

The diagram, Figure 3, shows that for the data set containing the 216 bacilli, labelled BOTH, 22 *m/z *intensities have been selected as relevant to discriminate between the different *Bacillus *species, whereas for the data set containing only vegetative cells and the data set containing only sporulated cells, 25 and 39 variables were selected respectively It is also clear that some ions (*m/z *57, 67, 74, 89, 104 and 136) were used by all classifiers irrespective of data partitioning.

### Species as classes: all bacilli

These experiments were aimed at classifying all the 216 Py-MS spectra from the bacilli into one of the seven possible species. Table 2 shows the confusion matrix for this classification when all 150 *m/z *ions were employed as well as the statistics related to these classifications. This table also details the results after GA variable selection down to the 22 ions highlighted in Figure 3. It is clear from the summary statistics (Table 2) that prediction was improved when only these 22 GA selected ions were used again highlighting the power of the feature selection performed by the GA-Bayesian network algorithm (GA-BN).

**Table 2:** Species: classification results for all bacilli (including vegetative and sporulated cases).

Confusion Matrices Species - all bacilli															
		**predicted class**													
		
		**all 150 *m*/*z *intensities**							**22 GA selected *m*/*z *intensities**						
			
		**sph**	**sub**	**lic**	**lat**	**cer**	**amy**	**meg**	**sph**	**sub**	**lic**	**lat**	**cer**	**amy**	**meg**

actual class	sph sub lic lat cer amy meg	29 3 1 0 0 1 1	1 24 1 0 0 3 0	0 0 21 0 0 0 3	0 1 0 23 1 0 0	0 0 0 1 14 0 0	0 14 7 0 0 26 0	0 0 0 0 15 0 26	30 3 1 1 3 0 6	0 32 0 0 0 3 0	0 0 22 0 0 1 0	0 0 1 23 0 0 0	0 0 0 0 25 0 0	0 7 6 0 0 26 0	0 0 0 0 2 0 24

	Statistics														
				
	class	TP	FP	precision	sensitivity				TP	FP	precision	sensitivity			
				
		all 150 *m*/*z *intensities							22 GA selected *m*/*z *intensities						
									
	sph	0.967	0.032	0.829	0.967				1	0.075	0.682	1			
	sub	0.571	0.029	0.828	0.571				0.762	0.017	0.914	0.762			
	lic	0.7	0.016	0.875	0.7				0.733	0.005	0.957	0.733			
	lat	0.958	0.01	0.92	0.958				0.958	0.005	0.958	0.958			
	cer	0.467	0.005	0.933	0.467				0.833	0	1	0.833			
	amy	0.867	0.113	0.553	0.867				0.867	0.07	0.667	0.867			
	meg	0.867	0.081	0.634	0.867				0.8	0.011	0.923	0.8			
				
	w. avg.	0.755	0.041	0.794	0.755				0.843	0.026	0.871	0.843			

Confusion matrices and summary statistics, w. avg. = weighted average.

**Figure 4:**
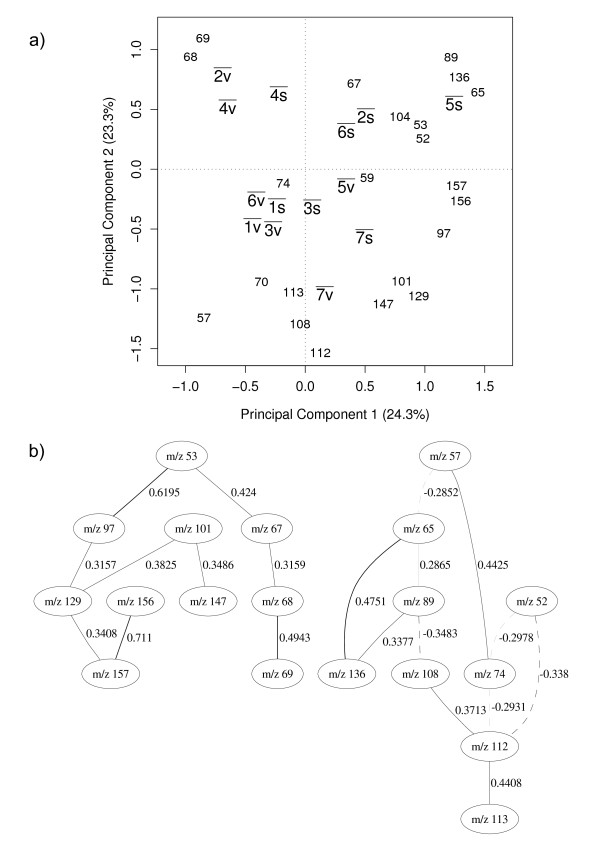
**Species all bacilli**. (a) principal component analysis discriminating among species (all bacilli). $\bar{\text{v}}=$ = vegetative, $\bar{\text{s}}\;\text{=}$ = sporulated, $\bar{1}=$ = *amyloliquefaciens*, $\bar{2}=$ = *cereus*, $\bar{3}=$ = *licheniformis*, $\bar{4}=$ = *megaterium*, $\bar{5}=$ = *sphaericus*, $\bar{6}=$ = *subtilis*, $\bar{7}=$ = *laterosporus*. The bar over the labels indicates that each point represents an average over all similar cases on the data set. $\bar{1\text{v}}\text{,}\ldots \text{,}\bar{7\text{v}}$ and $\bar{1\text{s}}\text{,}\ldots \text{,}\bar{7\text{s}}$ represent group centers for PC scores and the numbers (which represent *m/z *intensities) are PC loadings. (b) Bayesian network model. Solid lines indicate a positive correlation between nodes, dashed lines indicate negative correlation and the thickness of the line indicates the strength of the correlation. Therefore the thicker the line is the stronger the correlation. The number beside the line shows the partial correlation coefficient for that correlation.

Figure 4(a) shows the PC scores biplot for the average value of the classes and the associated PC loadings plot which shows the influence of the *m/z *intensities selected. This figure shows the classes and their relationship with the *m/z *intensities. The intensities *m/z 89*, m/z 136 and *m/z *65, for instance, are good discriminators for the species *B. sphaericus *$\left(\bar{5\text{s}}\right)$ when the *Bacillus *is sporulated but not as good when this species is in its vegetative state. Furthermore, the proximity of $\left(\bar{1\text{v}}\right)$ to $\left(\bar{1\text{s}}\right)$ and of $\left(\bar{\text{3v}}\right)$ to $\left(\bar{\text{3s}}\right)$ on the graph suggests that bacilli belonging to those respective species remain relatively similar regardless of their physiological state.

The Bayesian network model built on the 22 GA selected variables is shown in Figure 4(b). Interpretation of these relationships is difficult given the molecular nature of the ions in Py-MS (molecular ions are very rarely seen, rather complex pyrolysate and fragments thereof are detected but remain unidentified).

Despite this limitation of the data, rather than the BN, we see two separate sub-networks for species classifying when both spores and vegetative cells are included in the BN analysis. These relationships are not directly related to the differences between spores and vegetative cells, nor are these probabilistic relationships among variables wholly mirrored in PC scores (Figure 4(a)), and we see a mixture of selected ions from the different areas of PCA. For example, whilst *m/z *89, 136, 65 are highly positively correlated in both the BN and PCA the BN extends this network via a negative correlation to *m/z *108 and *m/z *57 which are located diagonally opposite in the PC loadings plot, but not *m/z *70 which is not used in the BN at all. In addition, despite *m/z *52 and 53 close relationship in PCA these are found to be separated in the BN. Thus this suggests that additional information could be generated from the BN when compared to PC loadings plots, and this will be an area of future work using ions of known origin.

### Species as classes: vegetative cells only

These experiments classified 108 mass spectra from the vegetative bacilli into one of the 7 possible species studied. Table 3 shows the confusion matrix for this classification as well as the statistics related to this classification for both the full Py-MS spectrum and for the 25 ions selected using GA, and in this case the data reduction has not led to a significant improvement in classification.

**Table 3:** Species: classification results for vegetative bacilli only.

Confusion Matrices Species - vegetative bacilli only															
		predicted class													
		
		all 150 *m*/*z *intensities							25 GA selected *m*/*z *intensities						
			
		sph	sub	lic	lat	cer	amy	meg	sph	sub	lic	lat	cer	amy	meg
actual class	sph sub lic lat cer amy meg	15 0 0 0 0 0 3	0 19 3 0 0 4 0	0 1 12 0 0 0 0	0 0 0 12 0 0 0	0 0 0 0 15 0 0	0 1 0 0 0 11 0	0 0 0 0 0 0 12	15 0 0 0 0 0 0	0 19 3 0 0 4 0	0 0 12 0 0 0 0	0 0 0 12 0 0 0	0 0 0 0 15 0 0	0 2 0 0 0 11 0	0 0 0 0 0 0 15

	Statistics														
				
	class	TP	FP	precision	sensitivity				TP	FP	precision	sensitivity			
				
		all 150 *m*/*z *intensities							25 GA selected *m*/*z *intensitie						s
									
	sph	1	0.032	0.833	1				1	0	1	1			
	sub	0.905	0.08	0.731	0.905				0.905	0.08	0.731	0.905			
	lic	0.8	0.011	0.923	0.8				0.8	0	1	0.8			
	lat	1	0	1	1				1	0	1	1			
	cer	1	0	1	1				1	0	1	1			
	amy	0.733	0.011	0.917	0.733				0.733	0.022	0.846	0.733			
	meg	0.8	0	1	0.8				1	0	1	1			
				
	w. avg.	0.889	0.023	0.902	0.889				0.917	0.019	0.926	0.917			

Confusion matrices and summary statistics, w. avg. = weighted average.

**Figure 5:**
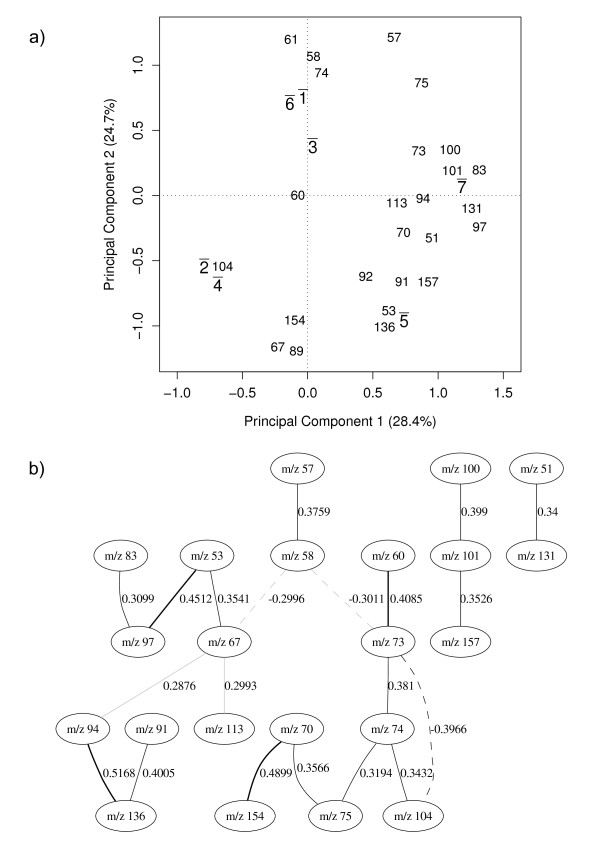
**Vegetative bacilli only**. (a) principal component analysis discriminating among species (vegetative bacilli only). $\bar{\text{v}}=$ = vegetative, $\bar{\text{s}}\;\text{=}$ = sporulated, $\bar{1}=$ = *amyloliquefaciens*, $\bar{2}=$ = cereus, $\bar{3}=$ = *licheniformis*, $\bar{4}=$ = *megaterium*, $\bar{5}=$ = *sphaericus*, $\bar{6}=$ = *subtilis*, $\bar{7}=$ = *laterosporus*. The bar over the labels indicates that each point represents an average over all similar cases on the data set. $\bar{1\text{v}}\text{,}\ldots \text{,}\bar{7\text{v}}$ and $\bar{1\text{v}}\text{,}\ldots \text{,}\bar{7\text{v}}$ represent group centers for PC scores and the numbers (which represent *m/z *intensities) are PC loadings. (b) Bayesian network model. Solid lines indicate a positive correlation between nodes, dashed lines indicate negative correlation and the thickness of the line indicates the strength of the correlation. Therefore the thicker the line is the stronger the correlation. The number beside the line shows the partial correlation coefficient for that correlation.

Figure 5(a) shows the PC scores biplot for the average value of the classes and the associated *m/z *intensities located in the PC loadings. This biplot was generated based on the 25 GA selected variables for this configuration of the data set and shows the locations of the species classes and their relationship with the *m/z *intensities. These relationships mirror very well the expected taxonomy of these bacilli as 4 groups are seen: (1) *B. subtilis*, *B. amyloliquefaciens *and *B. licheniformis*, (2) *B*. *cereus *and *B. megaterim*, (3*) B. sphaericus *and (4) *B*. *laterosporus*, and highlights that reduction of the data has not comprised the known relationships between these bacteria.

The Bayesian network model built on the 25 GA selected variables is shown in Figure 5(b).

### Species as classes: spores only

These experiments classified the Py-MS data from 108 sporulated bacilli into one of the 7 possible species studied. Table 4 shows the confusion matrix for these classifications as well as the statistics related to this classification for both the full Py-MS spectra and the 39 GA selected ions. In contrast to the model made from the vegetative cells, the reduced ions for the spores shows an improvement in the predictive ability for this model, which is encouraging considering that previous studies have suggested that the phenotype of *Bacillus *species on sporulation, as measured using Py-MS, becomes more similar and hence one would expect the predictions for spores to be worse than for vegetative cells [37].

**Table 4:** Species: classification results for sporulated bacilli only.

Confusion Matrices Species - sporulated bacilli only															
		predicted class													
		
		all 150 *m*/*z *intensities							39 GA selected *m*/*z *intensities						
			
		sph	sub	lic	lat	cer	amy	meg	sph	sub	lic	lat	cer	amy	meg
actual class	sph sub lic lat cer amy meg	14 0 0 0 1 0 0	0 21 0 0 0 1 0	0 0 14 0 0 0 0	0 0 0 11 0 0 1	1 0 0 1 14 0 0	0 0 1 0 0 14 0	0 0 0 0 0 0 14	15 0 0 1 0 0 0	0 21 0 0 0 0 0	0 0 14 0 0 0 0	0 0 0 11 0 0 0	0 0 0 0 15 0 0	0 0 1 0 0 15 0	0 0 0 0 0 0 15

	Statistics														
				
	class	TP	FP	precision	sensitivity				TP	FP	precision	sensitivity			
				
		all 150 *m*/*z *intensities							39 GA selected *m*/*z *intensities						
									
	sph	0.933	0.011	0.933	0.933				1	0.011	0.938	1			
	sub	1	0.011	0.955	1				1	0	1	1			
	lic	0.933	0	1	0.933				0.933	0	1	0.933			
	lat	0.917	0.01	0.917	0.917				0.917	0	1	0.917			
	cer	0.933	0.022	0.875	0.933				1	0	1	1			
	amy	0.933	0.011	0.933	0.933				1	0.011	0.938	1			
	meg	0.933	0	1	0.933				1	0	1	1			
				
	w. avg.	0.944	0.009	0.946	0.944				0.981	0.003	0.983	0.981			

Confusion matrices and summary statistics, w. avg. = weighted average.

**Figure 6:**
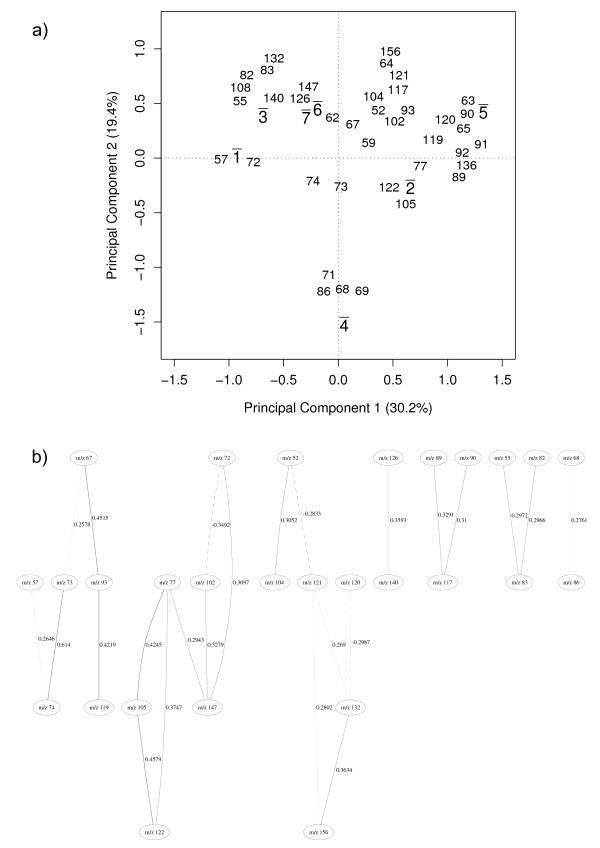
**Sporulated bacilli only**. (a) principal component analysis discriminating among species (sporulated bacilli only). $\bar{\text{v}}=$ = vegetative, $\bar{\text{s}}\;\text{=}$ = sporulated, $\bar{1}=$ = *amyloliquefaciens*, $\bar{2}=$ = cereus, $\bar{3}=$ = *licheniformis*, $\bar{4}=$ = *megaterium*, $\bar{5}=$ = *sphaericus*, $\bar{6}=$ = *subtilis*, $\bar{7}=$ = *laterosporus*. The bar over the labels indicates that each point represents an average over all similar cases on the data set. $\bar{1\text{v}}\text{,}\ldots \text{,}\bar{7\text{v}}$ = and $\bar{1\text{s}}\text{,}\ldots \text{,}\bar{7\text{s}}$ represent group centers for PC scores and the numbers (which represent *m/z *intensities) are PC loadings. (b) Bayesian network model. Solid lines indicate a positive correlation between nodes, dashed lines indicate negative correlation and the thickness of the line indicates the strength of the correlation. Therefore the thicker the line is the stronger the correlation. The number beside the line shows the partial correlation coefficient for that correlation.

Figure 6(a) shows the PC score biplot based on the 39 GA selected variables for the average value of the classes and the corresponding *m/z *intensities from the PC loadings matrix are also shown. In contrast to the same analysis from the vegetative cells, the taxonomic relationship between these bacilli whilst evident is more diffuse; that is to say there is overlap between *B. laterosporus *$\left(\bar{7}\right)$ and the *B. subtilis *like cluster $\left(\bar{1},\;\bar{3},\;\bar{6}\right)$. Figure 6(a) raises some other interesting observations. First, there is a suggestion that the cluster formed by *m/z *77, *m/z *105 and *m/z *122 could be related to the fragmentation pathway of benzoic acid as reported by [34,38]. Second, *m/z *77 and 105 are DPA related mass spectra [32] and their proximity to *B*. *cereus *on the PCA plot could indicate that *B*. *cereus *has a higher level of DPA than the other 6 *Bacillus *species studied in this work. This idea, however, seems to be contradictory. While some studies such as [39] suggest that *B. cereus *has in fact a high DPA level, higher than for *B. megaterium*, for instance, other studies such as [40,41] indicate the contrary. Although our PCA plot does seem to suggest that *B. cereus *has a high DPA level (agreeing with [39]), closer inspection of the data set tells a slightly different story. It reveals that the average values for *m/z *77 and *m/z *105 are higher for *B. megaterium *and *B*. *sphaericus *than they are for *B. cereus *(agreeing with [40,41]). Therefore, the reason why *m/z *77 and 105 are closer to *B. cereus *on the PCA plot may be due to the fact that *B. cereus *falls in-between *B. megaterium *and *B. sphaericus *on the plot. The Bayesian network model built on these 39 ions is shown in Figure 6(b) where the strong correlation involving *m/z *77, *m/z *105 and *m/z *122 is also highlighted.

### GA-Bayesian network vs. PLS-DA

To assess the power of the proposed GA-BN method as a feature selection technique we compare it to PLS-DA. Using exactly the same partitions of the data set assessed by the GA-BN algorithm, we applied PLS-DA to identify the most relevant features in each of those partitions. We then computed the new classification results based on those features selected by PLS-DA. Table 5 reports the following classification results: (1) the weighted true positive rate, (2) the total number of variables selected, and (3) the percentage of variables selected by PLS-DA that have also been selected by GA-BN, described as "overlap". The true positive rates from Table 5 show that variables selected by GA-BN separate the classes more accurately than the variables selected by PLS-DA, in all cases. In particular, the results suggest that as the number of classes increases, from 2 for physiological state to 7 to the other cases, the classification accuracy obtained by PLS-DA decreases noticeably. For the physiological state data partition there is a good agreement, 73.2%, between the features selected by GA-BN and PLS-DA. We expected PLS-DA to perform well on this partition of the data set because the PCA plot, Figure 2(a), suggests that this is a linearly separable system. By contrast, for the species vegetative only partition of the data set there is only a 1% agreement, and, although PLS-DA selected 41 variables more than GA-BN did, the PLS-DA model prediction accuracy was 13.0% lower.

**Table 5:** Classification results: comparison GA-Bayesian network vs. PLS-DA.

	True positive rate		# of variables selected		
		
Class type	GA	PLS-DA	GA	PLS-DA	Overlap
physiological state	99.5%	97.2%	17	15	73.2%
species both	84.3%	73.6%	22	47	12.7%
species veg. only	91.7%	78.7%	25	66	1.0%
species spo. only	98.1%	75.9%	39	11	45.4%

Overlap represents the percentage of variables selected by PLS-DA that have also been selected by GA-BN.

## Conclusions

In this study Py-MS data from a diverse group of *Bacillus *species were analysed using a novel approach of combining variable selection from GA with the probabilistic relationship inference from BN. This chemometrics-fusion approach was first used for the successful classification of spores *versus *vegetative biomass and subsequently the same data were used to identify the *Bacillus *species that was under analysis. The results of the physiological classification confirm that *m/z *105 which is a pyridine ketonium ion known to arise from DPA [8] plays a significant part in discriminating the spores from vegetative bacilli. Moreover, *m/z *77 was also selected which is known to be a fragment ion that results from pyridine ketonium [32]. A very parsimonious rule was constructed that only used four ions and had a 100% classification rate on the validation data. Taken together this shows that the GA-BN was able to discover novel biomarkers for spores and that these were validated by the know physiological differences that occur during sporulation [1]. Variable selection is an important aspect of any multivariate data analysis as it seeks to simplify a data set by reducing its dimensionality and identifying relevant underlying variables without sacrificing predictive accuracy. As a result for species classification the GA-BN significantly reduced the redundancy in the information provided by the variables actually used for prediction from 150 *m/z *to between 22-39 depending on the subset of the data analysed. As no single classifier works best on all given classification problems (see [42]), the present work designed a specific classification model for each partition of the data set analyzed. The results show that using significantly less *m/z *intensities, the classifiers obtained, on average, a better performance than the classifiers using all 150 *m/z *intensities available.

Taking the true positive (TP) rate as an example for analysing both spores and vegetative cells together the prediction from using 150 *m/z *to just 22 increased from 0.755 to 0.843. When only vegetative cells were analysed the TP rate was 0.889 for all 150 *m/z *and 0.917 for when the 22 GA selected variables were employed. By contrast, the TP rate increased from 0.944 to an impressive 0.981 when spores were analysed by Py-MS using either all 150 m/z or 39 selected ions respectively. This result indicates that not only are individual classifiers better than combining both spores and vegetative biomass, but that *Bacillus *speciation is better when spores are analysed. This is in contrast to what is expected from classical physiology studies and indicates that a lot more than just the production of DPA and specific proteins is occurring. This has implication for rapid analysis as one may be able to speciate the bacilli directly without the need for cultivation. Notwithstanding our results show that a hierarchical-like, or informed, classification of the bacilli into classes has shown a higher predictive accuracy than the classification without previous knowledge of physiological state.

The GA-BN algorithm has also outperformed a traditional classification method used in chemometrics, namely PLS-DA, in all cases tested. Although GA-BN did not always select the smallest subset of features, the classification accuracy indicated that it always selected the most relevant ones when compared to PLS-DA.

Bayesian networks explore two main characteristics of the target data set: associations among variables and the strength of these associations. The graphical model output from the GA-BN explicitly informs one about probabilistic associations. A conditional probability table stores the strength of the correlations given the associations displayed on the graphical model. Expert knowledge and statistical information can easily be introduced in BNs, as demonstrated in this study. BNs model the probability distribution of the problem domain and, therefore, can compute the predictive distribution on the outcomes of possible outputs.

In conclusion, we have developed a novel genetic algorithm-Bayesian network and demonstrated its implementation on a well described data set comprising pyrolysis mass spectra from a wide variety of different *Bacillus *species analysed both as vegetative cells and spores. The physiological assessment of these data reconfirmed that dipicolinic acid is a valuable biomarker for spore identification; whilst our hierarchical-informed classification structure showed excellent identification of the different species in the sporulated state, a finding that to our knowledge has not been shown before for Py-MS data.

## Authors' contributions

EC designed and implemented the code for the GA-BN algorithm, performed the statistical analysis of the data and drafted the manuscript. RG designed and performed the Py-MS analysis of the *Bacillus *data set, collected the data and collaborated with the interpretation of the results from the statistical data analysis and manuscript preparation. Both authors read and approved the final manuscript.

## Supplementary Material

Additional file 1 **The distribution of samples on the Bacillus data set**. This table shows the distribution of samples of the *Bacillus *Py-MS data set reported in this article. Click here for file

Additional file 2 **Bayesian network representing the lung cancer problem**. This figure shows a Bayesian network representing the lung cancer problem: L = low, H = high, T = true, F = false, Pos = positive and Neg = negative. Click here for file

Additional file 3 **Pseudocode for a generic greedy search algorithm**. Shows the pseudocode of a generic greedy search algorithm for learning Bayesian network structures. Click here for file
