# Supplementary material for: A genetic algorithm-Bayesian network approach for the analysis of metabolomics and spectroscopic data: application to the rapid identification of Bacillus spores and classification of Bacillus species
Source: BMC Bioinformatics. 2011 Jan 26;12:33. doi: 10.1186/1471-2105-12-33 (PMC3228543; doi:10.1186/1471-2105-12-33)
Supplement: Additional file 1 — The distribution of samples on the Bacillus data set. This table shows the distribution of samples of the Bacillus Py-MS data set reported in this article. [file 1471-2105-12-33-S1.DOC]

|  |  | vegetative | | sporulated | |
| --- | --- | --- | --- | --- | --- |
| bacteria | id. | quantity | replicates | quantity | replicates |
| *Bacillus sphaericus* | sph | 15 | 3 | 15 | 3 |
| *Bacillus subtilis* | sub | 21 | 3 | 21 | 3 |
| *Bacillus licheniformis* | lic | 15 | 3 | 15 | 3 |
| *Brevibacillus laterosporus* | lat | 12 | 3 | 12 | 3 |
| *Bacillus cereus* | cer | 15 | 3 | 15 | 3 |
| *Bacillus amyloliquefaciens* | amy | 15 | 3 | 15 | 3 |
| *Bacillus megaterium* | meg | 15 | 3 | 15 | 3 |
