# Supplementary material for: A genetic algorithm-Bayesian network approach for the analysis of metabolomics and spectroscopic data: application to the rapid identification of Bacillus spores and classification of Bacillus species
Source: BMC Bioinformatics. 2011 Jan 26;12:33. doi: 10.1186/1471-2105-12-33 (PMC3228543; doi:10.1186/1471-2105-12-33)
Supplement: Additional file 2 — Bayesian network representing the lung cancer problem. This figure shows a Bayesian network representing the lung cancer problem: L = low, H = high, T = true, F = false, Pos = positive and Neg = negative. [file 1471-2105-12-33-S2.PDF]

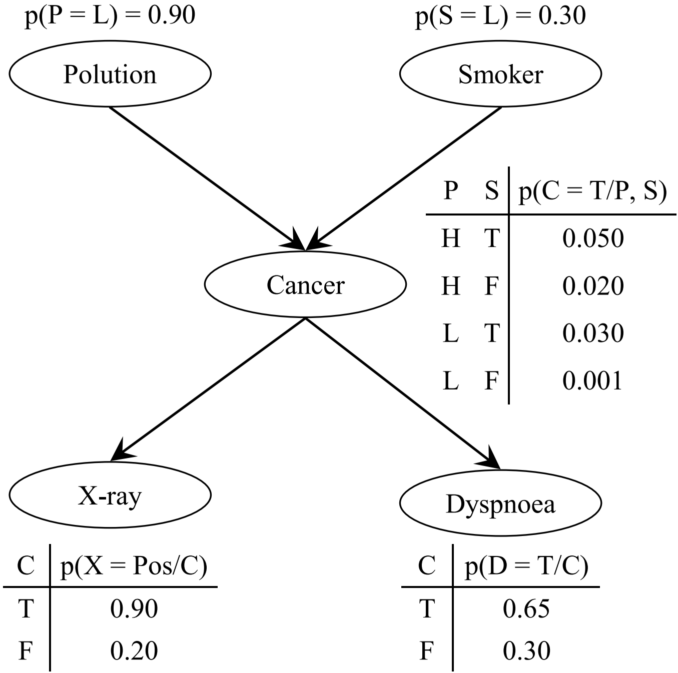

P	S	p(C = T/P, S)
H	T	0.050
H	F	0.020
L	T	0.030
L	F	0.001

C	p(X = Pos/C)
T	0.90
F	0.20

C	p(D = T/C)
T	0.65
F	0.30

Figure: Bayesian network representing the lung cancer problem:
L = low, H = high, T = true, F = false, Pos = positive and Neg = negative.
