# Supplementary material for: A genetic algorithm-Bayesian network approach for the analysis of metabolomics and spectroscopic data: application to the rapid identification of Bacillus spores and classification of Bacillus species
Source: BMC Bioinformatics. 2011 Jan 26;12:33. doi: 10.1186/1471-2105-12-33 (PMC3228543; doi:10.1186/1471-2105-12-33)
Supplement: Additional file 3 — Pseudocode for a generic greedy search algorithm. Shows the pseudocode of a generic greedy search algorithm for learning Bayesian network structures. [file 1471-2105-12-33-S3.PDF]

Algorithm Additional file 3: Pseudocode for a generic greedy search algorithm.

Require: Initialize an empty Bayesian network G containing n nodes (i.e., a BN with n nodes but no edges)

```
Evaluate the score of G: Score(G)
BEST = Score(G)
repeat
    FROM = 0
    TO = 0
    for i = 1 to n do
        for j = 1 to n do
            G' = G
            if i ≠ j then
                if there is no edge between the nodes i and j in G' then
                    Modify G': add an edge between the nodes i and j in G' such that i is a parent of j: (i → j)
                    if the resulting G' is a DAG then
                        if (Score(G') > BEST) then
                            BEST = Score(G')
                            FROM = i
                            TO = j
                        end if
                    end if
                end if
            end if
        end for
    end for
    if FROM > 0 then
        Modify G: add an edge between the nodes FROM and TO in G such that FROM is a parent of TO: (FROM → TO)
    end if
until FROM = 0
return G as the structure of the BN
```
